# Antibody contributes to heterosubtypic protection against influenza A-induced tachypnea in cotton rats

**DOI:** 10.1186/1743-422X-5-44

**Published:** 2008-03-20

**Authors:** Timothy M Straight, Martin G Ottolini, Gregory A Prince, Maryna C Eichelberger

**Affiliations:** 1 Department of Clinical Investigation, Brooke Army Medical Center, Fort Sam Houston, TX, USA; 2 Department of Medicine, Uniformed Services University of the Health Sciences, Bethesda, MD, USA; 3 Department of Pediatrics, Uniformed Services University of the Health Sciences, Bethesda, MD, USA; 4 Virion Systems Inc., Rockville, MD, USA; 5 CBER, Food and Drug Administration, Bethesda, MD, USA

## Abstract

**Background:**

Influenza virus infection or vaccination evokes an antibody response to viral hemagglutinin (HA) and neuraminidase (NA) surface glycoproteins, which results in immunity against influenza A viruses of the same HA and NA subtype. A heterosubtypic immune response that offers some protection against different influenza A subtypes has been suggested from epidemiologic studies in human influenza outbreaks, and has been induced in experimental animal models. Original studies of such cross-protection showed that cytotoxic T lymphocytes (CTL) protect H3N2-immune mice from a lethal H1N1 infection. More recent studies in mice demonstrate that antibodies also contribute to heterosubtypic immunity (HSI). We previously demonstrated that HSI in cotton rats (*Sigmodon hispidus*) is characterized by protection of H3N2-immune animals from influenza H1N1-induced increase in respiratory rate (tachypnea). Alternatively, H1N1-immune animals are protected from H3N2-induced tachypnea. The experiments described in this report were designed to elucidate the immune mechanism that prevents this very early sign of disease.

**Results:**

Our results show that cotton rats provided with H1N1-immune serum prior to challenge with an H3N2 virus were protected from influenza-associated tachypnea, with the degree of protection correlating with the antibody titer transferred. Immunization with an inactivated preparation of virus delivered intramuscularly also provided some protection suggesting that CTL and/or mucosal antibody responses are not required for protection. Antibodies specific for conserved epitopes present on the virus exterior are likely to facilitate this protection since prophylactic treatment of cotton rats with anti-M2e (the extracellular domain of M2) but not anti-nucleoprotein (NP) reduced virus-induced tachypnea.

**Conclusion:**

In the cotton rat model of heterosubtypic immunity, humoral immunity plays a role in protecting animals from influenza-induced tachypea. Partial protection against respiratory disease caused by different influenza A subtypes can be attained with either live virus administered intranasally or inactivated virus delivered intramuscularly suggesting that either vaccine regimen may provide some protection against potential pandemic outbreaks in humans.

## Background

Influenza A remains a major burden on mankind with annual epidemics of disease and continued potential for devastating pandemics such as that seen in 1918. Neutralizing antibodies that are specific for viral hemagglutinin (HA) and neuraminidase (NA) are induced following immunization with inactivated influenza vaccines and correlate with protective immunity against influenza strains of the same subtype. These specific antibodies do not offer protection against viruses that have a different HA and NA subtype, as noted in the vaccine failure in 1947 when an H1N1 virus emerged that was serologically distinct from the 1943 H1N1 strain used in the vaccine [1]. A more recent example of limited reactivity with a drifted influenza strain occurred in the 2003–2004 season when the vaccine contained an H3N2 virus that was antigenically distinct from newly circulating A/Fujian strain [2]. During this particular season it appeared that the live attenuated vaccine provided individuals with some protection against drifted strains of influenza [3], suggesting that a replicating virus administered intranasally is more likely to induce more broadly acting antibodies or cross-reactive cellular immune mechanisms that can act at the site of infection.

While immunity to influenza is primarily type and subtype-specific, epidemiologic evidence suggests that heterosubtypic immunity can be induced in man [4]. Retrospective studies that show a lower incidence of H2N2 influenza disease in persons previously infected with an H1N1 virus also support this idea [5]. However, the immune responses that correlate with protection of humans against infection with an influenza virus that is of a different subtype have not been characterized. Studies in influenza-infected mice suggest that multiple mechanisms may contribute to this type of protection. Traditionally, cell mediated immune mechanisms against conserved antigen targets have been considered responsible for a cross-protective immune response [6,7]. In contrast, more recent studies demonstrate a role for antibody in heterosubtypic immunity in mice [8,9]. These studies suggest that the magnitude of the immune response as well as the route of immunization is important in establishing antibody-mediated cross-protection.

The specificity of antibodies that provide protection against different influenza A subtypes are likely to be non-neutralizing, since antibodies that block HA-binding or inhibit NA activity are generally thought of as subtype-specific. These could include antibodies that recognize conserved portions of surface glycoproteins or antigens in the viral core. Examples of potential epitopes include a conserved peptide at the cleavage site of the influenza B HA molecule (this peptide has been used to induce immunity against influenza B strains that are antigenically distinct [10]) and the conserved extracellular peptide of M2 (M2e). It has been demonstrated that a monoclonal antibody with specificity for M2e inhibits influenza replication in mice [11] and that a M2e vaccine protects against lethal challenge with both H1N1 and H3N2 influenza A viruses in mice, and reduces shedding of viruses in ferrets [12].

We have used the cotton rat (*Sigmodon hispidus*) to study influenza pathogenesis and immunity. This unique model has the distinct advantage of exhibiting increased respiratory rate (tachypnea) following infection with influenza, a response that is dependent on virus dose and immune status. Respiratory rates are easily monitored by whole body plethysmography, making this a practical end-point to evaluate protection from influenza-induced respiratory disease or vaccine efficacy. We previously established that cotton rats can be used as a model to study heterosubtypic immunity against influenza A; animals exposed to one subtype of virus are protected from respiratory disease upon exposure to a different subtype of influenza A [13]. This protection is retained when animals are treated with steroid to inhibit the inflammatory response, suggesting that heterosubtypic immunity is not dependent on a recruited cellular response. In this report, we show that protection against influenza-induced tachypnea is transferred in serum from animals previously infected with an influenza virus of a different subtype, and examine the potential specificity of the cross-protective antibodies, as well as the route of immunization required to induce heterosubtypic immunity.

## Results

### Cross-protection is observed following the prophylactic transfer of serum from immunized animals to naïve cotton rats

Previous studies in our laboratory demonstrated that protection from respiratory disease was retained in immune animals after the administration of systemic steroids, which inhibited the acute inflammatory response following challenge with a heterosubtypic virus [14]. These results suggested that the heterosubtypic immune response was not mediated by recruited cells, but rather by local cells at the site of infection or cross-reactive antibodies. To further evaluate whether antibodies play a role in heterosubtypic immunity, we transferred serum from H1N1 or H3N2-immune cotton rats into naïve cotton rats 24 hr before intra-nasal (i.n.) challenge with 10^7 ^TCID_50_/100 g A/Wuhan/95, an H3N2 virus. Respiratory rates (RR) were measured 1 and 2 days later by whole body plethysmography.

**Figure 1:**
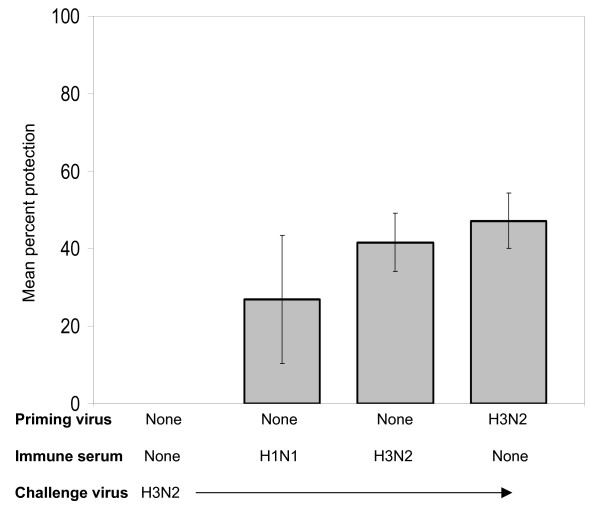
**Transfer of H1N1-immune serum protects recipient cotton rats against H3N2-induced tachypnea**. Mean percent protection (± SEM) is shown for animals that received H1N1 or H3N2-immune sera and were then challenged with an H3N2 virus, A/Wuhan/95. The immune sera were obtained from cotton rats previously infected with A/PR/8/34 (H1N1) or A/Wuhan/95 (H3N2). Peak respiratory rates were measured on day 2 after challenge and were used to calculate the mean percent protection from virus-induced tachypnea shown in the figure.

The group of animals that received H3N2-immune serum prior to viral challenge with H3N2 virus was significantly protected (p < 0.03) from the effects of respiratory disease compared to the group undergoing primary infection. The challenge group that was previously infected with the homotypic H3N2 virus was also protected from virus-induced tachypnea (p < 0.02). Passive transfer of H1N1-immune serum into 4 animals resulted in a strong trend toward protection, but the respiratory rates measured were not significantly different from the those measured in non-immune animals (p = 0.06). These results are presented in Fig. 1 as the mean percent protection from H3N2-induced tachypnea, with respiratory rates for day 2 post-challenge provided in the figure legend.

**Figure 2:**
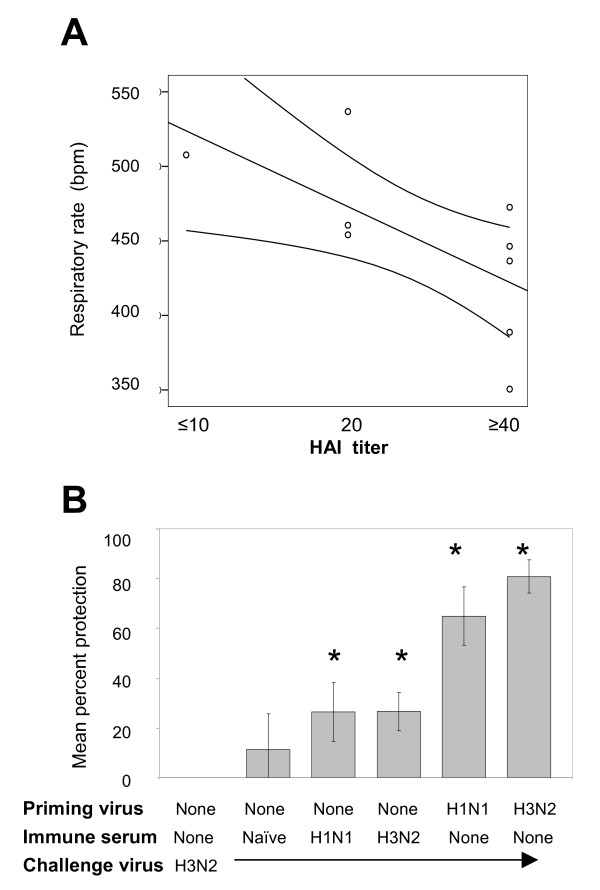
**Correlation of protection against tachypnea and HAI titer after passive transfer of heterosubtypic immune sera**. Respiratory rates (breaths per minute) and serum HAI titers are shown for individual animals in A. These animals were challenged with A/Wuhan/95 (H3N2) after receipt of H1N1-immune sera. The best fit line and 95% confidence intervals are displayed in the figure. The Spearman's correlation coefficient was -0.71 (p < 0.02). Percent protection from tachypnea for groups of animals that received immune sera before H3N2 challenge is shown in B. These groups included animals that did not receive serum, or groups that received from naïve, H1N1-immune or H3N2-immune animals. The mean protection was calculated using results from animals that had HAI titer ≥ 40 following serum transfer. Results are also shown for control groups that were immune to the homotypic or heterosubtypic virus at the time of challenge. Percent protection of different groups were compared by Mann-Whitney test, with statistical significant differences (p < 0.05) with the group experiencing primary infection in the absence of immune serum marked with a *.

Variation in the degree of protection in recipients of H1N1-immune serum suggested that the i.p. inoculation of serum may not always transfer an equal amount of antibody into the circulation. To assess the quantity of antibody transferred in each animal, we measured hemagglutination inhibition (HAI) titers in the serum of recipients 12 hr after intraperitoneal (i.p.) transfer of immune sera. The degree of protection from tachypnea correlated with the recipient's pre-challenge HAI titer (Fig. 2A), with Spearman's correlation coefficient of -0.71 (p < 0.02). In general, animals with higher HAI titers demonstrated lower RR than recipients of naïve serum. In subsequent passive transfer studies, only animals with an HAI titer of 40 or greater were considered successful transfer recipients and an HAI titer ≥ 40 was a prerequisite for including individual animal results in the data analysis. Data collected 2 days post-infection in one such experiment are displayed in Fig. 2B, showing mean percent protection calculated from the mean respiratory rates provided for each animal group in the figure legend. Statistical analysis showed that the RR of animals receiving either heterosubtypic (A/PR/8/34)-immune or homologous (A/Wuhan/95)-immune-serum were significantly less than naïve animals undergoing primary infection (p < 0.03 and p < 0.01 respectively). Previous studies show that tachypnea is close to resolution by day 4 post-infection and therefore respiratory rates were not measured at this time point. At this late time point, animals did not exhibit any gross difficulty in breathing, and did not have increased histopathology, suggesting that there was no exacerbation of disease. Animals administered non-immune serum prior to transfer did not differ significantly from animals undergoing primary disease (p = 0.24).

### Neutralizing antibodies in serum of immune cotton rats are subtype specific

To evaluate whether antibodies with hemagglutination inhibition activity contribute to this *in vivo *cross-protection, we examined the ability of serum from H1N1-immune animals (the same pool of serum that had been used in the transfer study) to inhibit agglutination of red blood cells by A/Wuhan/95 (H3N2). The pooled serum had an HAI titer of 640 against A/PR/8/34 but <10 against A/Wuhan/95 (Table 1). This lack of cross-reactivity is expected, indicative of a subtype-specific neutralizing antibody response. To evaluate whether the antibodies that neutralize virus replication are truly subtype-specific in this model, we also determined the amount of antibody required to inhibit replication of H1N1 or H3N2 viruses in MDCK cells. The tissue-culture neutralizing titer for H1N1-immune serum in this assay was 1600 against A/PR/8/34 and <100 against A/Wuhan/95. Because complement component C1q can enhance the activity of antibodies [15], the neutralization assay was also performed in the presence of complement. Addition of C1q increased the neutralizing antibody titer to 3200 but did not change the specificity of the inhibition. A pool of serum from A/Wuhan/95-immune animals showed similar subtype specificity, with a titer of 200 against A/Wuhan/95 that increased to 800 in the presence of complement. Even in the presence of complement, this serum did not inhibit A/PR/8/34 replication at the lowest dilution of antibody used (1/100). Antibodies that inhibited NA activity were also subtype specific; the NA inhibition (NI) titer of H1N1-immune serum that had been used in transfer studies was 80 against A/PR/8/34 and no detectable inhibition was measured against the N2 activity of A/Wuhan/95. The NI titer of H3N2-immune serum was 320 against A/Wuhan/95 and there was no detectable inhibition against the NI activity of A/PR/8/34.

**Table 1:** Subtype-specific antibody responses are evident in sera from A/PR/8/34(H1N1) and A/Wuhan/95(H3N2)-infected animals.

	*Antibody titer as measured by*^a^							
	*HAI*		*NI*		*Neutralization*		*Neut + C1q*	
				
*Serum source*	*H1N1*	*H3N2*	*H1N1*	*H3N2*	*H1N1*	*H3N2*	*H1N1*	*H3N2*
Naïve serum	<10	<10	0	0	<100	<100	<100	<100
H1N1-immune	640	<10	80	0	1600	<100	3200	<100
H3N2-immune	<10	160	0	320	<100	200	<100	800

^a^Standard hemagglutination inhibition (HAI), neuraminidase inhibition (NI) and neutralization (neut) assays in the absence as well as presence of complement factor C1q were performed as described in Materials and Methods. Viruses used for these assays were A/PR/8/34 (H1N1) and A/Wuhan/359/95 (H3N2) that had been used to infect the cotton rats that were the source of this serum pool. Animals were boosted several times by rechallenging them with the same virus before serum was collected. The lowest dilution of serum used in the HAI assay was 1/10 and therefore no inhibition of agglutination is recorded as a titer of < 10. The lowest dilution of serum used in the neutralization assay was 1/100 and therefore no neutralization is recorded as a titer of < 100.

### Protection from virus-induced tachypnea is achieved by prophylactic administration of antibodies specific for viral M2 but not viral NP

**Figure 3:**
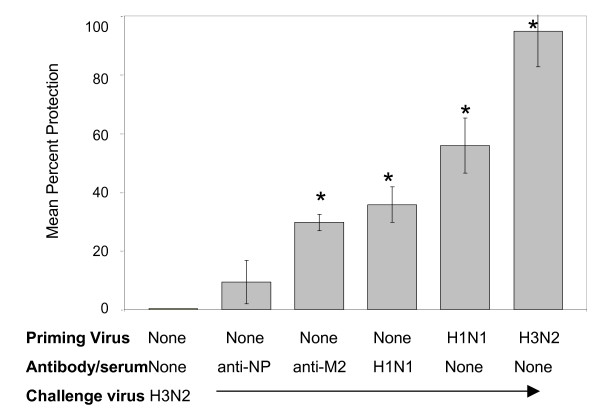
**Antibodies specific for M2 but not NP protect against influenza-induced tachypnea**. Groups of 6 animals were inoculated i.p. with 100 μg monoclonal antibody (anti-M2 or anti-NP) prepared in saline solution 24 hr before infection with A/Wuhan/95 (H3N2). Control groups of animals underwent passive transfer of 0.5 ml (i.p.) of serum from H1N1-immune animals, or were either infected with the same H3N2 virus or A/PR/8/34 (H1N1) virus 28 days earlier. The percent protection was calculated from RR measured by whole body plethysmography. Groups of animals that had RR statistically different (p < 0.05) from animals undergoing primary influenza infection are designated in the figure with an *.

Antibody with specificity for M2e provides protection against influenza A replication in mice, and therefore has the potential to play a role in reducing tachypnea following infection of cotton rats. To test whether this is the case, groups of cotton rats were treated (i.p. inoculation) with 100 μg monoclonal antibody specific for either influenza nucleoprotein (NP) or M2e 6 hr before infection with A/Wuhan/95 (10^7 ^TCID_50_/100 g). Four animals were used in each group. Cotton rats that received anti-M2e, but not anti-NP prior to challenge were subsequently protected from tachypnea an (p < 0.04, and p < 0.48, respectively). These results are shown in Fig. 3.

### Heterosubtypic immunity is observed following immunization with UV-inactivated virus that is delivered intramuscularly, and does not require immunization with live virus

Since our cotton rat model of heterosubtypic immunity was established using live virus to vaccinate cotton rats i.n., we examined the ability of inactivated virus to protect animals from virus-induced tachypnea. We also determined whether mucosal immunization was essential to induce heterosubtypic immunity by comparing protection in animals that have been vaccinated i.n. and intramuscularly (i.m.). Since protection against tachypnea was successfully transferred in serum from animals that were immune to heterosubtypic virus, we expected that transudated rather than local mucosal antibodies were responsible for this protection. The A/PR/8/34 virus was inactivated by exposure to UV-light and its inability to replicate verified by titration in MDCK cells. Equivalent amounts of virus (10^7 ^TCID_50_/100 g) were used to inoculate groups of animals (4 animals per group) i.n. and i.m. with live or inactivated virus. Serum samples were obtained from all animals 2 weeks after immunization to evaluate immune responses by measuring HAI titers.

**Figure 4:**
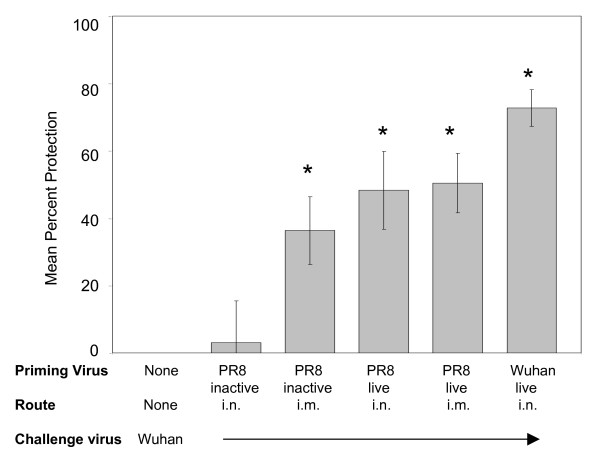
**Intramuscular immunization with inactivated H1N1 virus protects against H3N2-induced tachypnea**. Groups of animals (4 cotton rats per group) were inoculated with the equivalent of 10^7 ^TCID_50 _A/PR/8/34 (H1N1) per 100 g. Both live and UV-inactivated virus preparations were inoculated intranasally (i.n.) or intramuscularly (i.m.). Animals in groups immunized with inactivated virus were boosted at week 3 and 6. HAI titers of serum samples obtained by retro-orbital bleed 2 weeks following the final immunization are included in the text. All groups were challenged 10 weeks following the first immunization with A/Wuhan/95 (H3N2). Control groups included naïve animals that provided baseline RR, naïve animals infected with A/Wuhan/95 for the first time, and A/Wuhan/95-challenged H3N2-immune cotton rats. RR were measured by whole body plethysmography and the percent protection from tachypnea calculated for each animal. Protection that was statistically greater than non-immune animals (p < 0.05) is marked with an *.

As expected, exposure to live virus administered i.n. resulted in greater HAI titers than exposure to inactivated virus. Groups of cotton rats that were immunized with the inactivated H1N1 virus were therefore boosted 3 times with this virus preparation at 3 week intervals. At the time of intranasal virus challenge with the heterosubtypic A/Wuhan/95 virus, there was no inhibition of A/Wuhan/95 agglutination of chicken red blood cells. The serum HAI geometric mean titers (GMT) against A/PR/8/34 varied substantially in each of the groups (4 animals per group): 11 following i.n. immunization with inactivated virus; 28 following i.m. immunization with inactivated virus; 100 following i.n. inoculation with live virus; 82 following i.m. inoculation with live virus. The HAI titer in sera of cotton rats infected once with A/Wuhan/95 that served as a homotypic control group, was 57. As expected, this serum did not inhibit agglutination with the H1N1 virus. Protection from influenza-induced tachypnea was observed in the groups of animals immunized i.m. with either live or inactivated virus preparations (Fig. 4), indicating that a local immune response was not required to provide cross-protection. Protection against tachypnea was not observed in the group of animals immunized intranasally with inactivated virus. This group had the lowest HAI titer, suggesting that insufficient titers of cross-protective antibodies had been attained under these conditions.

## Discussion

Heterosubtypic immunity in man has been suggested from epidemiologic studies of human outbreaks of influenza A [4,5,16]. Identification of the immune components necessary for a heterosubtypic immune response will be critical in the development of more broadly protective vaccines effective against influenza A virus. Both antibodies and cytotoxic T cells have been implicated in cross-protective immune responses in murine models of influenza infection, where the most often used end-point is mortality.

In the cotton rat model, we previously demonstrated that respiratory rate can be used as a measure of disease severity [13]. Protection from tachypnea is observed in cotton rats immunized with one subtype of influenza A virus and subsequently challenged with another subtype, demonstrating a heterosubtypic immune response. This protection persists despite inhibition of the recruited memory response [14]. The studies presented in this report show that protection is mediated by humoral immunity since passive transfer of immune serum from H1N1-immune animals is able to transfer components necessary for protection from H3N2-induced tachypnea. Protection correlates with HAI titer. While the HAI titer is a measure of a subtype-specific antibodies, it also reflects the total amount of antibody successfully administered during the passive transfer and is therefore likely to correlate with the amount of cross-reactive antibodies present in the serum. These antibodies are most likely specific for conserved epitopes of influenza A, and may include antibodies with specificity for NP, M2e or conserved HA peptides. Non-neutralizing HA-specific antibodies that may contribute to B cell-dependent, heterosubtypic protection against lethal infection by avian H5N1 influenza have been measured in the convalescent sera of mice [9]. While there is good evidence that M2-specific antibodies are induced following infection [17], we were unable to measure anti-M2 titers in our cotton rat serum samples in an ELISA using M2e peptide to coat the plates. The poor sensitivity of this type of assay has been reported and it is known that functional M2e-specific antibodies are best detected using a cell-based expression system [17]. While we do not know the fine specificities of antibodies present in convalescent cotton rat sera, our results show that M2e-specific but not NP-specific monoclonal antibodies can contribute to protection from influenza virus-induced tachypnea.

Further studies are needed to evaluate how antibodies contribute to cross-protection. They may reduce the amount of virus that can attach to cells by directing FcR-positive macrophages to the pathogen for uptake and degradation. A role for macrophages in heterosubtypic immunity is supported by the studies of Sambhara et al. [18]. Alternatively, cross-protective antibodies may work in conjunction with NK cells as demonstrated for protection of mice by M2-specific antibodies [19]. Our finding of antibody-mediated cross-protection against tachypnea in the cotton rat model is an important step toward recognition that this type of response is not limited to mice, and is therefore likely to be present in other animal species, including man.

Our results show that heterosubtypic immunity can be induced by vaccination with either live or inactivated virus that is administered intramuscularly. These results differ from those reported by Tumpey et al. [8] and Takada et al. [20] that show heterosubtypic protection in mice following vaccination with intranasal but not intramuscular-delivery of an inactivated virus vaccine. This latter failure to protect against challenge in mice is likely to reflect the relatively weak responses induced following parental immunization. In our studies three intra-muscular administrations of inactivated virus resulted in HAI titers similar to those obtained following infection; this vaccination regimen was sufficient for heterosubtypic protection supporting the idea that a mucosal IgA response is not necessary for this protection.

Increased respiratory rate is a single facet of influenza disease, and while an antibody-mediated mechanism protects against virus-induced tachypnea in cotton rats, it is likely that other immune mechanisms contribute to protection against other signs of disease. This may include cytokines that have antiviral activity or activate macrophages, and cytotoxic T lymphocytes that play a role in eradicating infected cells. Influenza vaccines that induce a broad range of mechanisms are likely to offer the most effective protection against all influenza A viruses, an important consideration in the development of vaccines designed to induce immunity against highly virulent H5N1 strains with potential for pandemic spread. Our results support the idea that antibodies specific for conserved epitopes play a role in protection from influenza induced disease and are therefore likely to contribute to vaccine efficacy, particularly when HA and NA components are poorly matched with circulating influenza A viruses.

## Conclusion

Passive transfer of serum from H1N1-immune cotton rats provides protection against H3N2-induced tachypnea even though the antiserum lacked subtype cross-reactivity in standard HAI, NI or neutralization assays. Since recent studies demonstrate that antibodies contribute to heterosubtypic immunity in mice, these studies in a second animal model support the idea that this mechanism may provide some immune protection against respiratory disease in humans. Such heterosubtypic protection was observed in animals immunized with either live or inactivated virus preparations delivered intranasally or intramuscularly respectively, demonstrating that current human influenza vaccine strategies are likely to induce some heterosubtypic immunity. While the specificity of antibodies that provide cross-protection is have not been fully characterized, our results demonstrate that monoclonal antibodies to M2e but not NP provide some protection against virus-induced tachypnea. This supports the idea that antibodies to conserved epitopes on the surface of the virion or infected cell contribute to heterosubtypic immunity. It is important to establish that similar responses are induced following human vaccination and contribute to vaccine efficacy. Our future studies will therefore characterize the quality and quantity of antibodies that provide heterosubtypic immunity so that tests can be designed to evaluate these responses following human vaccination.

## Materials and methods

### Cotton rats

Male and female inbred *Sigmodon hispidus *were obtained from a breeding colony maintained at Virion Systems, Inc., Rockville, MD. Animals were seronegative for adventitious viruses. Prior to infection, they were also seronegative for influenza A as tested by HAI assay. Animals were used at 6–12 weeks of age in protocols that follow federal regulations and were approved by the Institutional Animal Care and Use Committee. Animals were sacrificed by CO_2 _asphyxiation for the collection of tissue samples.

### Viruses

Influenza A/Wuhan/359/95 (A/Wuhan/95), an H3N2 virus, was grown in MDCK cells at Novavax Inc. (Rockville, MD), resulting in a virus stock solution of 10^8 ^TCID_50_/ml. Tissue culture-adapted influenza A/PR/8/34 (H1N1) was obtained from ATCC, and was grown in a monolayer of MDCK cells resulting in a viral titer of 10^8 ^TCID_50_/ml. Virus was stored at -70°C, and thawed immediately prior to use. Aliquots of A/PR/8/34 that were exposed to UV-light did not contain any infectious virus.

### Measurement of respiratory rates

Respiratory rates (RR) were measured by unrestrained whole body flow plethysmography (Buxco Electronics Inc., Wilmington, NC) as described previously [13]. After calibration of the 2-chamber apparatus (designed to hold adult rats), one cotton rat was placed in each chamber and airway measurements were continuously recorded over a 5-minute period. The mean respiratory rate over the entire 5-minute period was calculated. Data from each group are presented as mean breaths per minute (+/- standard error) or as the percent protection from tachypnea calculated as: 100 - {100 × [(RR_experimental group _- RR_uninfected_)/(RR_primary infection_-RR_uninfected_)]}.

### Hemagglutination inhibition (HAI) assay

Serum was treated with receptor destroying enzyme (RDE) overnight and then serially diluted in PBS. One volume (25 μl) of each dilution was mixed with 1 volume of A/Wuhan/95 containing 4 hemagglutinating units of virus in a U-bottomed 96-well plate. After 30 min incubation at room temperature, 2 volumes of a 0.5% suspension of chicken red blood cells (CBT Farms, Chestertown, MD) were added, the suspension gently mixed and left to settle at room temperature for 30 min. Agglutination was read and the inverse of the last dilution that inhibited agglutination assigned as the titer.

### Neuraminidase inhibition (NI) assay

Two-fold dilutions of serum (50 ul per well) were mixed with an equal volume of virus. The amount of virus added provided a signal 10-fold greater than background. Substrate labeled with fluorochrome, 2,4-methylumbelliferone-N-acetyl neuraminic acid (MU-NANA), was then added (100 μl of a 20 μM solution) as previously described for measurement of NA activity [21]. After 1 hr incubation at room temperature the reaction was stopped by addition of 100 ul 0.1 M glycine, pH 10.7 containing 25% EtOH. Fluorescence (365 excitation, 460 emission, 0.1 sec per well) was read on a Victor 3 (Perkin Elmer). The inverse of the last dilution of virus that resulted in at least 50% reduction of NA activity was recorded as the NI titer.

### Virus neutralization assay

Serial dilutions of serum were made in DMEM, starting with a 1/100 dilution. An equal volume (100 μl) of virus (200 TCID_50_/ml) was added and the mixture incubated at room temperature for 15 minutes. A portion (100 μl) of the virus-antibody mixture was transferred to duplicate MDCK cell monolayers in 96 well plates that had been washed 3 times with serum-free medium. After 1 hr incubation at 37°C, an equal volume of DMEM containing 1% bovine serum albumin and TPCK-treated trypsin (5 μg/ml) was added to each well, and the plates were returned to the incubator. On day 3 of incubation, the supernatants were discarded and the monolayers fixed and stained with crystal violet. Neutralization titers were assigned as the inverse of the last dilution that inhibited the viral cytopathic effect in both of the duplicate wells. The neutralization assay was also performed in the presence of complement, with addition of 25 μl of a solution of C1q (5 μg/ml) to each well of the tissue culture plate.

### Experimental design

Anesthetized animals were immunized by intranasal (i.n.) administration of 10^7 ^TCID_50 _virus per 100 grams of animal as previously described [22]. This dose of virus is not lethal to cotton rats and corresponds to approximately 100 μl total volume (a 6 week old animal weighs approximately 100 g). This volume is sufficient to deliver the inoculum into the lower respiratory tract, resulting in virus replication in lungs, trachea and nasal tissue. Groups of animals that were not immunized, or immunized with either A/Wuhan/95 (H3N2) or A/PR/8/34 (H1N1) were challenged with the H3N2 virus four weeks later. Sera for transfer studies were obtained from animals never exposed to influenza (naïve control), or exposed to either H3N2 or H1N1 viruses at 3-week intervals 3 times previously. The serum from individual animals in each group were pooled and transferred (0.5 ml per animal) by intra-peritoneal injection 24 hr prior to i.n. challenge with virus. Twelve hr before challenge, retro-orbital bleeds were performed on the recipient animals to obtain sera to measure HAI titers. Respiratory rates were measured by whole body plethysmography.

### Statistical Analysis

Mean respiratory rates (RR) were compared between groups by non-parametric Kruskal-Wallis and Mann-Whitney tests. All analyses were performed using SPSS (version 13.0) statistical software. *P*-values of <0.05 were considered statistically significant.

## Competing interests

The authors declare that they have no financial competing interests. The opinions or assertions contained in this report are the private views of the authors and are not to be construed as reflecting the views of the Uniformed Services University, U.S. Department of the Army, U.S. Department of the Air Force, the U.S. Department of Defense, or the Food and Drug Administration.

## Authors' contributions

TMS and MCE designed and executed experiments, analyzed data, and wrote the manuscript. MGO provided substantial input to study design and manuscript preparations. GAP gave final approval for publication. All authors read and approved the final manuscript.
